# Malpositions of the Colon

**Published:** 1892-08

**Authors:** Thomas Walley

**Affiliations:** Principal Royal Veterinary College, Edinburgh


### MALPOSITIONS OF THE COLON.

By Thomas Walley, M.R.C.V.S.,
*Principal Royal Veterinary College, Edinburgh.*

To those who, like myself, have made the subject of malpositions of the colon a study, the articles published in the December issue of the Journal are of more than passing interest. Before alluding particularly to these, it may be as well that I should refer to my own position in connection with the matter. In the earliest days of my professional career the subject of bowel lesions in the horse was to me an interesting and fascinating one, but it was not until the year 1861—while acting as assistant to the late Mr. Edward Stanley of Birmingham—that I formulated in my own mind any theory in reference to the nature of the serious, and so often fatal, bowel affections to which the horse was, *par excellence*, the subject. The views I formulated were the outcome of many hours' (by day and by night) patient watching, repeated rectal explorations, and numerous *post-mortem* examinations.

Practical expression of these views was first given by me in the year 1870, while assisting in the practice of Mr. Lawson at Manchester, and that on the occasion of a *post-mortem* examination which I made on the body of a horse whose death was due to torsion of the colon. It so happened that at the autopsy in question several of the city practitioners were present, and the whole of them expressed the opinion that the lesions made manifest by opening the abdomen were those of enteritis. I was enabled to show, distinctly, that they were the result of complete torsion with vascular strangulation, and I then made the statement, often reiterated since, that not one in twenty of the so-called cases of enteritis were in reality of that nature, but, on the contrary, that they were cases of malposition or torsion of the colon.

In 1875 I read a paper on the subject at a meeting of the Liverpool Veterinary Medical Association, in which I pointed out that such lesions were of far more frequent occurrence than was enteritis, and that in many cases a successful diagnosis could be made. A few months prior to the reading of this paper, Mr. Elam, of Liverpool, had expressed views on the subject identical with my own.

So satisfied did I feel that most of the cases of bowel affections in the horse which had a fatal termination were of the nature of malpositions, that I did not hesitate to express my views with some decision to the members of my class when lecturing on bowel derangements, and in the April number of *The Journal of Comparative Medicine and Surgery* for 1888, I formulated these views more precisely.

I there divided colic malpositions into—

(1) Simple Displacement,
(2) Version,
(3) Torsion, the first consisting of a throwing of one of the curvatures of the colon (usually the pelvic) out of its normal position to the opposite side of the abdominal cavity; the second consisting in (*a*) *Lateral Version*, in which one of the colic curvatures is turned partially on its own axis, constituting partial torsion—(*b*) *Anteversion*, in which the pelvic flexure is thrown in a forward direction, doubled on itself—(*c*) *Retroversion*, in which either the diaphragmatic or the supra-sternal flexure is thrown in a backward direction on itself; the third consisting in a *complete twist* of the bowel (sometimes double twist) on its own axis. I further pointed out that the gravity of these malpositions was in a high degree regulated by the extent of interference with the circulation (vascular strangulation) of the blood-vessels of the involved portion of intestine. In speaking of the differential diagnosis of these cases, I directed attention not only to the systemic symptoms presented (to which I may say other writers on the subject do not appear to attach the importance they deserve), but in particular to the physical signs detectable by rectal exploration, *e. g.*—"(*a*) The presence in the pelvic cavity, or on one side of the lumbar region, of a resilient tumour, the fundus of which is presented *towards* the hand of the explorer, and which owes its origin to decomposition (and, as a result thereof, liberation of gas) of the contents of the involved knuckle of intestine—this tumour being permanent and rapidly returning to its original position if displaced by pressure with the hand." "(*b*) The tense, twisted condition of the muscular bands of the colon, the direction taken by which indicates with accuracy the direction of the twist or displacement."

I am quite prepared to acknowledge that in speaking of the twisted condition of the muscular bands of the colon I referred more particularly to cases of torsion, and while perfectly familiar with the conditions described by Jelkman, Moller and Malkmus, I never attached the same amount of importance to them as I did, and as I now do, to twisting of the colic bands in a horizontal or longitudinal direction. How far I was justified in assuming this position may be judged by several cases which have lately come under my observation, and to which I shall presently refer. Before doing this, however, I wish to direct attention to a statement of Moller's, to the effect that he attaches more importance to the bands of the colon than he does to the tense and stretched condition of the mesentery referred to by Jelkman. The relative importance of the two conditions may be a matter of opinion, but I confess that from my own point of view the latter is of more importance than the former, as it is more constantly present and constitutes the greater element of danger. It will be observed that the word "torsion" is employed by the authorities quoted, in connection with the particular form of malposition which they describe, but none of them seems to differentiate between partial torsion—lateral version—and complete; from the course of their cases and the general symptoms presented there is little or no doubt in my own mind that in nearly all, if not in all the cases related, the condition actually in existence was one of partial torsion only, and without any material strangulation of the vessels.

In the short space of three weeks after reading the articles alluded to in a series of seven cases of bowel lesion occurred in my practice, consisting of (*a*) one case of rotary torsion of the small intestines; (*b*) three cases of complete torsion of the colon; (*c*) three cases of colic (lateral) version, or displacement of the colon from left to right; the first four were fatal, the last three, which were, in every sense, identical in character with those described by the authors already referred to, recovered. Naturally, I was much interested in these cases, especially in the first of the three, and made an unusually careful rectal exploration; not only so, but in the first case I followed out faithfully the directions given by Jelkman for the purpose of effecting a reduction of the displacement, persevering in my efforts until I was afraid to make any further attempt at reduction. During the course of my manipulations a small quantity of gas escaped per anus, and I thought my work would be crowned with success; such, however, was not the case. I had previously ordered the administration of a dose of physic (as is my wont in all cases of colic in which the pain does not pass off after the exhibition of a colic draught), and I contented myself with the abstraction of blood (also a common practice of mine in cases of this kind where the pulse seems to warrant it), the application of hot cloths to the abdomen, the administration of morphia and other anodynes, as occasion required, and the occasional use of enemas.

In the course of forty-eight hours the bowels reacted, and the horse was rapidly restored to his original condition.

In the other two cases, after ascertaining the condition of matters, I did not make any prolonged attempt at reduction, nor did I bleed; otherwise the treatment was the same as in the first case, and the result equally satisfactory.

The course of these cases was only a repetition of many similar ones which have come under my observation and in which, I

candidly confess, the prognosis was very unfavorable, the result eminently satisfactory.

In reference to the mode in which reduction is brought about in this class of cases, opinions may differ; I incline to the view that it results, firstly, from sudden spasmodic contraction of the involved portion of bowel, and secondly, from the changes in position assumed by the patient while laboring under the effects of pains. In all cases of colic I have for a very long period been in the habit of giving strict injunctions that the patient should not be allowed to roll; frequently, however, when I have reason to believe that some form of displacement has taken place I give instructions that rolling may be allowed, and I have more than once seen sudden relief follow this act.—*The Journal of Comparative Pathology and Therapeutics.*

